# Waist to Height Ratio Is an Independent Predictor for the Incidence of Chronic Kidney Disease

**DOI:** 10.1371/journal.pone.0088873

**Published:** 2014-02-12

**Authors:** Keiichi Odagiri, Isagi Mizuta, Makoto Yamamoto, Yosuke Miyazaki, Hiroshi Watanabe, Akihiko Uehara

**Affiliations:** 1 Department of Clinical Pharmacology and Therapeutics, Hamamatsu University School of Medicine, Higashi-ku, Hamamatsu, Japan; 2 Yamaha Health Care Center, Naka-ku, Hamamatsu, Japan; 3 Department of Mental Health, Institute of Industrial Ecological Sciences, School of Medicine, University of Occupational and Environmental Health, Yahatanishi-ku, Kitakyushu, Japan; University of Milan, Italy

## Abstract

**Objective:**

Obesity is a risk factor for chronic kidney disease (CKD) and cardiovascular disease. The association between waist to height ratio (WheiR) and CKD is unclear. This study evaluated the association between WheiR and CKD.

**Design and Methods:**

In this longitudinal cohort study, 4841 Japanese workers (3686 males, 1155 females) 18 to 67 years of age in 2008 were followed up until 2011. CKD was defined as an estimated glomerular filtration rate of <60 mL/min/1.73 m^2^ (by the Modification of Diet in Renal Disease equation for Japanese) or dipstick proteinuria (≥1+). Cox proportional hazards models were used to examine the relationship between WheiR and development of CKD.

**Results:**

A total of 384 (7.9%) participants (300 men and 84 women) were found to have new CKD. The incidence of CKD was 13.7, 24.2, 37.9 and 43.7 per 1000 person-years of follow-up in the lowest, second, third and highest quartiles of WheiR, respectively. After adjustment for potential confounders, the adjusted hazard ratios (95% confidence interval) for CKD were 1.00 (reference), 1.23 (0.85, 1.78), 1.59 (1.11, 2.26) and 1.62 (1.13, 2.32) through the quartiles of WheiR, respectively. WheiR had a significant predictive value for the incidence of both proteinuria and low estimated glomerular filtration rate. After subdivision according to gender, the relationship between WheiR and the incidence of CKD was statistically significant in the unadjusted model. However, after adjusting for potential confounders, WheiR was significantly associated with the incidence of CKD in females, whereas it was not significant in males.

**Conclusions:**

WheiR, which is commonly used as an index of central obesity, is associated with CKD. There was a significant gender difference in the relationship between CKD and WheiR.

## Introduction

Chronic kidney disease (CKD) is a relatively common disorder. Patients with CKD have an increased risk of end-stage renal disease. Furthermore, CKD is also related to both a higher prevalence of cardiovascular disease and premature death [1], [2]. The estimated proportion of patients with CKD in the general population is approximately 10% in the United States, 7% in the United Kingdom, 9−10% in Asian countries and 19% in Japan [3]–[7]. The prevalence of CKD has been increasing significantly with time [8], [9]. Therefore, screening, diagnosis and treatment of CKD are important issues in preventing disease onset and progression [10], [11].

A recent study reported that obesity is a primary risk factor for cardiovascular disease, diabetes mellitus, hypertension and metabolic syndrome, and that these risk factors are associated with CKD [12]. Many authors consider that abdominal obesity is superior to body mass index (BMI) in predicting cardiometabolic risk, and visceral fat is known to have high metabolic and inflammatory activity in comparison to subcutaneous fat [13]–[17]. Waist to height ratio (WheiR) is recognized as an effective index for identifying central obesity, which relates to cardiometabolic risk factors [18]. One meta-analysis has demonstrated that WheiR is statistically superior to both waist circumference (WC) and BMI in the evaluation of cardiometabolic risk [19]. However, few studies have demonstrated an association between WheiR and CKD. Thus, we hypothesized that WheiR was associated with the incidence of CKD. We tested this hypothesis by investigating the 3-year incidence of CKD in Japanese workers.

## Materials and Methods

### Ethics Statement

The Institutional Review Board waived the need for informed consent from the participants because this research was a retrospectively observational analysis, and the identifying information was not included in the collected data. The ethics committee of Yamaha Health Insurance Society approved this study.

### Study Design and Participants

We designed a retrospective longitudinal cohort study to investigate the relation between WheiR and the incidence of newly diagnosed CKD in Japanese employees at general health checkups at the Yamaha Health Care Center, Hamamatsu, Japan. Most of the participants were employees of manufacturing companies in Hamamatsu, Japan. The Industrial Safety and Health Law in Japan requires employers to conduct annual health examinations of all employees. Our study utilized routinely collected data from these checkups. Because many participants were expected to have repeated examinations, we took advantage of this opportunity to conduct a follow-up study on CKD.

A total of 7025 participants (5337 men and 1688 women) who underwent general health checkup between January 2008 and December 2008 were enrolled in the study. Participants' ages ranged from 18 to 67 years (mean 41.7±11.1 years). After enrollment, the participants were followed up by general health checkups every year. We excluded 2184 participants who were already diagnosed with CKD (n = 545), lacked some laboratory data (n = 194) and had missing data for health behaviors (n = 3) at baseline. In addition, we excluded the participants who were not followed up in 2009 (n = 1442). Overall, the current analysis comprised 4841 participants (3686 men and 1155 women) with complete data on all the health behaviors and general health checkups. Among those participants, 673 were lost to follow up. However, we had no information on the causes of losing to follow up. The mean follow-up period was 2.7±0.7 years (range, 0.2−3.9), 12,926 person-years.

### Data Collection and Measurements

#### Measurements

After an overnight fast, blood and urine samples were obtained to measure blood levels of routine medical checkup tests: The complete blood count was measured automatically in a Sysmex XE 2100 equipment (Roche Diagnostics, Kobe, Japan). Fasting plasma glucose (FPG), triglycerides (TG), creatinine (Crt) and low-density lipoprotein (LDL) cholesterol were measured enzymatically. High-density lipoprotein (HDL) cholesterol was measured using the precipitation method. Hemoglobin A1c (HbA1c) was measured using the latex agglutination method. Uric acid (UA) was measured using uricase/POD method, Liver function tests including γ-glutamyltransferase (GGT), aspartate aminotransferase (AST), alanine aminotransferase (ALT) were measured using LDH-UV method. Simple qualitative urinalyses were performed with a dipstick on flesh, mid-stream urine collection in the morning, as a single control test. Chemical measurements were all performed at a local laboratory (Medic, Hamamatsu, Japan). Systolic blood pressure (SBP) and diastolic blood pressure (DBP) were measured with the participants in a sitting position. Height and body weight were measured with the participants wearing lightweight clothing. WC was measured at navel level in a standing position with light expiration.

#### Questionnaire interview

Participants were all required to fill out a self-administered standardized questionnaire of “Specific Health Checkups” in Japan, which included information about lifestyle factors and medical history, including smoking status (never, former or current smoker), alcohol drinking behavior (non/rare, sometimes, everyday), regular exercise habit (exercising at least 2 days/week, at least 30 minutes) and antihypertensive, antidiabetic, antidyslipidemic and antihyperuricemic medications. Three health behaviors − smoking status, alcohol intake and regular exercise − were selected as study variables from self-administered questionnaires.

### Definitions

BMI was calculated as weight in kilograms divided by the square of height in meters. Estimated glomerular filtration rate (eGFR) was calculated as eGFR (mL/min/1.73 m^2^) = 194×Crt ^−1.094^ × age ^−0.287^ in men, and 194× creatinine ^−1.094^ × age ^−0.287^×0.739 in women according to the criteria of the Japanese Society of Nephrology [20]. WheiR was calculated by dividing the WC by body height. Low eGFR was defined as eGFR < 60 mL/min/1.73 m^2^. Proteinuria was defined as dipstick proteinuria (≥1+). CKD was diagnosed on the basis of low eGFR and/or proteinuria which were present in single test.

### Follow-up and End Points

Participants were followed from the starting point until December 31, 2011. The primary end point of this analysis was an incidence of new CKD. Study participation was considered to be complete if the participant had an occurrence of the primary end point, was unable to be followed further because of loss of follow-up, or had been followed through at least to December 31, 2011. The exposure time was calculated as the time between the first visit to our institution and the point of either incidence of new CKD or the date of the last study visit if that came first.

### Statistical Analysis

All the data were expressed as the mean ± standard deviation (SD) (parametrically distributed values) or median (interquartile range) (non-parametrically distributed values) of the indicated numbers. We divided the study population into gender-adjusted quartiles of the distribution of WheiR. Differences among the quartiles of WheiR were examined by one-way analyses of variance (ANOVA) (parametrically distributed values) or Kruskal-Wallis test (non-parametrically distributed values). The polynomial contrast test and Kendall tau rank correlation coefficient (Kendall's tau-b) were used for trend analysis of numerical and categorical variables. Categorical variables were compared among the quartiles of WheiR by the chi-square test. Cox proportional-hazards models were used to calculate hazard ratios and 95% confidence intervals (CI) for the comparison of event rates while controlling for potential confounders. The potential confounders were selected from clinical variables and lifestyle factors associated with CKD (Table 1). Receiver operating characteristic (ROC) analyses were used to compare the predictive function of different methods in identifying individuals with Obesity, and Hanley-McNeil test was used to examine difference between area under the curves (AUCs). A p-value < 0.05 was regarded as statistically significant. The computations were performed using SPSS programs (version 11.0 J; SPSS Inc., Chicago, IL, USA).

**Table 1:** Definitions of potential confounders selected from clinical variables and lifestyle factors associated with CKD.

Hypertension	Blood pressure >140/>90 mmHg and/or antihypertensives use
Diabetes mellitus	HbA1c ≥6.5%, and/or FPG ≥126 mg/dL and/or antidiabetes use
Increased LDL cholesterol	LDL cholesterol ≥140 mg/dL and/or antidyslipidemics use
Decreased HDL cholesterol	HDL cholesterol < 40 mg/dL (in men), < 50 mg/dL (in women)
Hypertriglyceridemia	TG≥150 mg/dL
Hyperuricemia	UA≥7.0 mg/dL and/or antihyperuricemics use
Preserved eGFR	eGFR≥80 ml/min/1.73 m^2^
Urine occult blood	Dipstick hematuria (≥1+)

HbA1c, hemoglobin A1c; LDL cholesterol, low-density lipoprotein cholesterol; HDL cholesterol, high-density lipoprotein cholesterol; eGFR, estimated glomerular filtration rate

## Results

### Baseline Clinical Characteristics

The upper limits of the lowest (Q1), second (Q2), and third quartiles of WheiR (Q3) were set at 0.44, 0.47 and 0.50 for males, and 0.43, 0.46 and 0.50 for females, respectively. The mean values for quartiles Q1, Q2, Q3 and Q4 of WheiR were 0.41±0.02, 0.46±0.01, 0.49±0.01 and 0.54±0.03 for males, and 0.41±0.02, 0.45±0.01, 0.48±0.01 and 0.55±0.05 for females, respectively.

Tables 2 and 3 show the participants' baseline demographic and clinical characteristics and health behaviors. There were significant associations between WheiR and age, body weight, BMI, SBP, DBP, ALT, AST, GGT, LDL cholesterol, HDL cholesterol, TG, FPG, HbA1c, UA, red blood cell count (RBC), hematocrit (Hct), hemoglobin (Hb), platelet counts (Plt) and leukocyte count (WBC), but there was no relationship between WheiR and Crt. Furthermore, significant positive trends for quartiles of WheiR were found for body weight, BMI, SBP, DBP, ALT, LDL cholesterol, FPG, HbA1c, UA, RBC, Hct, Hb, Plt and WBC, while significant negative trends were shown for HDL cholesterol and eGFR (all p < 0.001 with the polynomial contrast test). As shown in Table 3, increasing WheiR was associated with higher proportion of hypertension, diabetes mellitus, increased LDL cholesterol, decreased HDL cholesterol, hypertriglyceridemia, and hyperuricemia (all p < 0.001 with Kendall's tau-b), while the proportion of urine occult blood and regular exercise did not associate with quartiles of WheiR.

**Table 2:** Baseline Characteristics of the Study Participants^a^.

	Q1	Q2	Q3	Q4	p value
Participants, n	1210	1210	1211	1210	
Age, median (interquartile range) (year)	34 (18–67)	40 (18–66)	44 (18–63)	49 (18–67)	<0.001
Male gender	922 (76.2%)	921 (76.1%)	922 (76.1%)	921 (76.1%)	1.000
Height (cm)	168.7±7.9	167.5±7.8	166.7±7.6	165.1±8.6	<0.001
Body weight (kg)	54.7±7.7	59.2±8.8	62.8±9.1	70.5±12.6	<0.001
Body Mass Index (kg/m^2^)	19.1±1.5	20.9±1.7	22.4±1.8	25.6±3.1	<0.001
Systolic blood pressure (mmHg)	109.9±11.4	113.1±12.5	116.1±13.4	120.7±13.8	<0.001
Diastolic Blood Pressure (mmHg)	68.1±8.9	71.2±9.8	73.6±10.1	76.9±10.1	<0.001
Aspartate aminotransferase (IU/L)	20.9±7.8	21.6±7.0	22.5±8.1	26.0±14.4	<0.001
Alanine aminotransferase (IU/L)	15 (3–135)	18 (4–107)	20 (5–165)	25 (5–282)	<0.001
Gamma glutamyltransferase (IU/L)	20 (7–381)	23 (7–539)	26 (7–523)	34 (7–521)	<0.001
LDL Cholesterol (mg/dL)	101.6±25.5	115.0±28.4	122.6±29.5	132.2±30.2	<0.001
Triglyceride (mg/dL)	63 (22–424)	78 (25–613)	90 (23–942)	111 (26–1155)	<0.001
HDL-cholesterol (mg/dL)	70.7±16.7	67.1±17.3	63.2±16.7	58.0±15.1	<0.001
Creatinine (mg/dL)	0.82±0.13	0.82±0.14	0.82±0.14	0.81±0.13	0.057
eGFR (ml/min/1.73 m^2^)	83.2±12.3	80.3±12.6	78.2±12.6	78.3±12.2	<0.001
Fasting Plasma Glucose (mg/dL)	94.2±11.0	97.3±16.1	98.5±12.1	104.3±20.3	<0.001
HbA1c (%)	5.2±0.4	5.3±0.5	5.3±0.5	5.5±0.7	<0.001
Uric Acid (mg/dL)	5.3±1.2	5.5±1.3	5.7±1.3	5.9±1.4	<0.001
Red blood cell count (10^4^/µL)	484.3±41.1	485.4±42.6	488.9±41.7	498.4±41.3	<0.001
Hemoglobin (g/dL)	14.5±1.4	14.6±1.4	14.6±1.5	15.0±1.5	<0.001
Male	15.0±1.0	15.2±0.9	15.2±1.0	15.6±1.0	<0.001
Female	12.8±1.0	12.8±1.2	12.7±1.4	13.0±1.4	0.032
Hematocrit (%)	43.9±3.6	44.2±3.7	44.3±3.9	45.1±3.9	<0.001
Platelets (10^4^/µL)	23.9±4.9	24.8±5.2	25.0±5.4	25.7±6.1	<0.001
Leukocyte count (10^9^/L )	5.6±1.5	5.9±1.5	5.1±1.5	6.4±1.7	<0.001

a Data are expressed as n (%) or mean ± SD or median (interquartile range)

Q1, lowest quartiles; Q2, second quartiles; Q3, third quartiles; Q4, quartiles; HDL cholesterol, high-density lipoprotein cholesterol; LDL cholesterol, low-density lipoprotein cholesterol; eGFR, estimated glomerular filtration rate; HbA1c, hemoglobin A1c

**Table 3:** Clinical Characteristics of the Study Participants^a,b^.

		Q1	Q2	Q3	Q4	p value
Urine occult blood		55 (4.5%)	61 (5.0%)	44 (3.6%)	59 (4.9%)	0.345
Hypertension		34 (2.8%)	66 (5.5%)	105 (8.7%)	221 (18.3%)	<0.001
Diabetes mellitus		9 (0.7%)	28 (2.3%)	24 (2.0%)	102 (10.8%)	<0.001
Increased LDL cholesterol		104 (8.6%)	229 (18.9%)	329 (27.2%)	482 (39.8%)	<0.001
Decreased HDL cholesterol		28 (2.3%)	36 (3.0%)	59 (4.9%)	104 (8.6%)	<0.001
Hypertriglyceridemia		29 (2.4%)	128 (10.6%)	202 (16.7%)	351 (29.0%)	<0.001
Preserved eGFR		684 (56.5%)	538 (44.5%)	473 (39.1%)	435 (36.0%)	<0.001
Hyperuricemia		1101 (91.5%)	1039 (85.9%)	999 (82.5%)	924 (76.4%)	<0.001
Smoking status						<0.001
	Never smokers	747 (61.7%)	681 (56.3%)	652 (53.8%)	645 (53.3%)	
	Former smokers	175 (14.5%)	201 (16.6%)	252 (20.8%)	268 (22.2%)	
	Current Smokers	288 (23.8%)	328 (27.1%)	307 (25.4%)	297 (24.5%)	
Alcohol drinking behavior						<0.001
	Non/Rare	523 (43.2%)	438 (36.2%)	450 (37.2%)	507 (41.9%)	
	Sometimes	488 (40.3%)	473 (39.1%)	456 (37.7%)	430 (35.5%)	
	Every day	199 (16.4%)	299 (24.7%)	305 (25.2%)	273 (22.6%)	
Regular exercise		256 (21.2%)	278 (23.0%)	260 (21.5%)	270 (22.3%)	0.696

a Data are expressed as n (%).

b Definitions of these confounding factors are shown in Table 1.

WheiR, waist to height ratio; Q1, lowest quartiles; Q2, second quartiles; Q3, third quartiles; Q4, quartiles; LDL cholesterol, low-density lipoprotein cholesterol; HDL cholesterol, High-density lipoprotein cholesterol; eGFR, estimated glomerular filtration rate.

### The Incidence of CKD in Relation to Quartiles of WheiR

A total of 384 (7.9%) participants including 300 men and 84 women received a new diagnosis of CKD during the study period, and 45, 79, 122 and 138 diagnoses were received in Q1, Q2, Q3 and Q4 of WheiR, respectively. The overall incidence of CKD was 29.7 per 1000 person-years. A graded relationship existed between WheiR and the incidence of CKD (Table 4).

**Table 4:** Incidence of CKD in Relation to Quartiles of Waist to height ratio (WheiR).

Quartile of WheiR	Incidence (/1000 person-years)	Unadjusted hazards ratio(95% CI)	p value	Adjusted hazards ratio (95% CI)^a^	p value	Adjusted hazards ratio (95% CI)^b^	p value
Q1	13.7	(reference)		(reference)		(reference)	
Q2	24.2	1.75 (1.29, 2.52)	0.003	1.23 (0.85, 1.78)	0.275	0.99 (0.65, 0.66)	0.963
Q3	37.9	2.78 (1.98, 3.91)	<0.001	1.59 (1.11, 2.26)	0.011	1.15 (0.73, 1.80)	0.547
Q4	47.3	3.31 (2.36, 4.65)	<0.001	1.62 (1.13, 2.32)	0.009	1.14 (0.67, 1.91)	0.633
p for trend			<0.001		0.004		0.464

a (model 2) Adjusted for age, gender, smoking status, alcohol drinking behavior, regular exercise, hypertension, increased low-density lipoprotein cholesterol, decreased High-density lipoprotein cholesterol, hypertriglyceridemia, hyperuricemia, diabetes mellitus, urine occult blood, hemoglobin and preserved eGFR at baseline.

b (model 3) Adjusted for model 2 plus body mass index. Cox proportional-hazards models were used to calculate hazard ratios and 95% confidence intervals. Definitions of these confounding factors are shown in Table 1.

WheiR, waist to height ratio; CI, confidence interval, Q1, lowest quartiles; Q2, second quartiles; Q3, third quartiles; Q4, highest quartiles

In the unadjusted analysis, Cox proportional-hazards models revealed that the risk of CKD rose with each increasing quartile of WheiR. In a test for interaction between the quartiles of WheiR and follow-up time, there was no violation of the proportional-hazards assumption. In the multivariable analysis, the significant graded association between WheiR and the hazards ratio for CKD persisted after adjustment for age, gender, smoking status, alcohol drinking behavior, regular exercise, hypertension, increased LDL cholesterol, decreased HDL cholesterol, hypertriglyceridemia, hyperuricemia, urine occult blood, hemoglobin and preserved eGFR at baseline (Table 4). However, after further adjustment for BMI, WheiR was no longer a risk factor for the incidence of CKD.

Because significant negative trend was also shown for eGFR at baseline though the quartiles of WheiR (Table 2), we next performed further assessment for the relationship among eGFR, WheiR and a risk of CKD to eliminate the effect of baseline eGFR level. We divided the study participants into gender-, and eGFR at baseline (eGFR ≥80 ml/min/1.73 m^2^ or <80 ml/min/1.73 m^2^) - adjusted quartiles of the distribution of WheiR, and then, we calculated the hazards ratios for the incidence of CKD. In participants with decreased eGFR (eGFR < 80 ml/min/1.73 m^2^), the unadjusted hazards ratio (HR) (95% confidence interval (CI)) for the incidence of CKD was 1.00 (reference), 1.67 (1.17, 2.39, p = 0.005), 2.64 (1.76, 3.45, p < 0.001) and 2.39 (1.70, 3.36, p < 0.001) though the quartiles of re-classified WheiR, respectively. After adjustment for age, gender, smoking status, alcohol drinking behavior, regular exercise, hypertension, increased LDL cholesterol, decreased HDL cholesterol, hypertriglyceridemia, hyperuricemia, urine occult blood and hemoglobin, this significant graded association between re-classified WheiR and the hazards ratio for CKD persisted (adjusted HR (95%CI); 1.00 (reference), 1.41 (0.98, 2.03, p = 0.064), 1.81 (1.28, 2.58, p = 0.001) and 1.61 (1.12, 2.34, p = 0.011) though the quartiles of re-classified WheiR, respectively). However, in participant with preserved eGFR (eGFR ≥80 ml/min/1.73 m^2^), these associations were not observed (adjusted HR (95%CI); 1.00 (reference), 0.71 (0.21, 2.37, p = 0.575), 0.81 (0.25, 2.59, p = 0.721) and 2.12 (0.73, 6.16, p = 0.036) though the quartiles of re-classified WheiR, respectively).

### The Incidence of Low eGFR and Proteinuria in Relation to Quartiles of WheiR

Table 5 shows the unadjusted and adjusted hazard ratios for low eGFR and proteinuria. In the unadjusted model, there was a significant graded association between WheiR and the incidence of both low eGFR and proteinuria, and these relationships persisted after adjustment for age, gender, smoking status, alcohol drinking behavior, regular exercise, hypertension, increased LDL cholesterol, decreased HDL cholesterol, hypertriglyceridemia, hyperuricemia, urine occult blood, hemoglobin and preserved eGFR at baseline. After further adjustment for BMI, WheiR associated with the incidence of neither proteinuria nor low eGFR.

**Table 5:** Incidence of Proteinuria and Low eGFR in Relation to Quartiles of Waist to height ratio (WheiR).

	Quartile of WheiR	Unadjusted hazards ratio (95% CI)	p value	Adjusted hazards ratio (95% CI)^a^	p value	Adjusted hazards ratio (95% CI)^b^	p value
Low eGFR	Q1		(reference)		(reference)		(reference)
	Q2	2.00 (1.35, 2.94)	0.001	1.33 (0.90, 1.98)	0.152	1.05 (0.68, 1.34)	0.82
	Q3	3.10 (2.15, 4.47)	<0.001	1.64 (1.12, 2.39)	0.011	1.15 (0.71, 1.85)	0.579
	Q4	3.36 (2.34, 4.85)	<0.001	1.54 (1.04, 2.27)	0.03	1.04 (0.60, 1.81)	0.881
	p for trend		<0.001		0.029		0.811
Proteinuria	Q1	(reference)	(reference)	(reference)			
	Q2	0.56 (0.16, 1.91)	0.356	0.57 (0.18, 2.00)	0.387	0.50 (0.13, 1.98)	0.323
	Q3	1.16 (0.42, 3.19)	0.778	1.21 (0.42, 3.49)	0.724	0.89 (0.20, 3.95)	0.88
	Q4	3.28 (1.40, 7.67)	0.006	2.68 (1.01, 7.10)	0.047	1.58 (0.29, 8.57)	0.594
	p for trend		0.001		0.011		0.366

a (model 2) Adjusted for age, gender, smoking status, alcohol drinking behavior, regular exercise, hypertension, increased low-density lipoprotein cholesterol, decreased high-density lipoprotein cholesterol, hypertriglyceridemia, hyperuricemia, diabetes mellitus, urine occult blood, hemoglobin and preserved eGFR at baseline.

b (model 3) Adjusted for model 2 plus body mass index. Cox proportional-hazards models were used to calculate hazard ratios and 95% confidence intervals. Definitions of these confounding factors are shown in Table 1.

WheiR, waist to height ratio; CI, confidence interval; Q1, lowest quartiles; Q2, second quartiles; Q3, third quartiles; Q4, highest quartiles.

### Gender Differences in the Relationship between the Incidence of CKD and Quartiles of WheiR

Next, we investigated the association between gender, WheiR and the incidence of CKD. Table 6 shows the unadjusted and adjusted hazard ratios for CKD for males and females. In the unadjusted analysis, a graded relationship existed between WheiR and the risk of CKD in both males and females. However, after adjusting for potential confounders including BMI, WheiR was significantly associated with the incidence of CKD in females, whereas it was not significant in males.

**Table 6:** Incidence of CKD in Relation to Quartiles of Waist to height ratio (WheiR) Stratified by Gender.

Gender	Quartile of WheiR	Unadjusted hazards ratio (95% CI)	p value	Adjusted hazards ratio (95% CI)a	p value	Adjusted hazards ratio (95% CI)b	p value
Male	Q1	(reference)	(reference)	(reference)			
	Q2	1.42 (0.96, 2.11)	0.081	0.94 (0.63, 1.41)	0.781	0.64 (0.40, 1.03)	0.063
	Q3	2.18 (1.52, 3.14)	<0.001	1.18 (0.80, 1.73)	0.412	0.70 (0.42, 1.17)	0.168
	Q4	2.73 (1.91, 3.90)	<0.001	1.25 (0.84, 1.86)	0.269	0.71 (0.39, 1.30)	0.267
	p for trend		<0.001		0.111		0.746
Female	Q1	(reference)	(reference)				
	Q2	6.40 (1.90, 21.63)	0.003	5.31 (1.55, 18.16)	0.008	5.06 (1.45, 17.59)	0.011
	Q3	11.42 (3.49, 37.36)	<0.001	9.24 (2.76, 30.91)	<0.001	8.12 (2.25, 29.75)	0.001
	Q4	11.49 (3.51, 36.42)	<0.001	7.18 (2.12, 24.19)	0.002	5.77 (1.41, 23.62)	0.015
	p for trend		<0.001		0.001		0.041

a Adjusted for age, smoking status, alcohol drinking behavior, regular exercise, hypertension, increased low-density lipoprotein cholesterol, decreased high-density lipoprotein cholesterol, hypertriglyceridemia, hyperuricemia, diabetes mellitus, urine occult blood, hemoglobin and preserved eGFR at baseline.

b (model 3) Adjusted for model 2 plus body mass index. Cox proportional-hazards models were used to calculate hazard ratios and 95% confidence intervals. Definitions of these confounding factors are shown in Table 1.

WheiR, waist to height ratio; CI, confidence interval; Q1, lowest quartiles; Q2, second quartiles; Q3, third quartiles; Q4, highest quartiles

### Comparison of the Predictive Values of CKD in Each Surrogate Maker of Obesity

We also investigated the relationship between other markers of obesity including WC and BMI, and the incidence of CKD. To assess these relations, we divided the study participants into gender-adjusted quartiles of the distribution of WC and BMI. Table 7 shows the unadjusted and adjusted hazards ratios for CKD in each surrogate marker. In the unadjusted analysis, a graded relationship existed between both WC and BMI, and the risk of CKD. In the multivariable analyses, the significant graded association between these markers and the hazards ratio for CKD persisted after adjustment for potential confounders.

**Table 7:** Comparison of the Predictive Values of CKD in Each Surrogate Maker of Obesity.

Gender	Marker	Quartile of WheiR	Unadjusted hazards ratio (95% CI)	p value	Adjusted hazards ratio (95% CI)^a^	p value
Overall	Waist circumference	Q1	(reference)		(reference)	
		Q2	1.91 (1.33, 2.73)	<0.001	1.41 (0.98, 2.02)	0.068
		Q3	2.47 (1.75, 3.48)	<0.001	1.47 (1.03, 2.09)	0.033
		Q4	3.06 (2.19, 4.30)	<0.001	1.67 (1.16, 2.39)	0.006
		p for trend		<0.001		0.009
	Body mass index	Q1	(reference)		(reference)	
		Q2	2.08 (1.46, 2.97)	<0.001	1.58 (1.10, 2.25)	0.013
		Q3	2.66 (1.88, 3.75)	<0.001	1.80 (1.27, 2.57)	0.001
		Q4	2.90 (2.07, 4.08)	<0.001	1.79 (1.25, 2.56)	0.001
		p for trend		<0.001		0.003
Males	Waist circumference	Q1	(reference)		(reference)	
		Q2	1.67 (1.12, 2.48)	0.012	1.14 (0.77, 1.72)	0.507
		Q3	2.33 (1.61, 3.38)	<0.001	1.34 (0.90, 1.97)	0.139
		Q4	2.57 (1.78, 3.73)	<0.001	1.38 (0.92, 2.07)	0.122
		p for trend		<0.001		0.088
	Body mass index	Q1	(reference)		(reference)	
		Q2	2.25 (1.51, 3.35)	<0.001	1.68 (1.12, 2.52)	0.012
		Q3	2.63 (1.78, 3.90)	<0.001	1.78 (1.19, 2.67)	0.005
		Q4	2.82 (2.82, 4.15)	<0.001	1.79 (1.18, 2.70)	0.006
		p for trend		<0.001		0.019
Females	Waist circumference	Q1	(reference)		(reference)	
		Q2	3.45 (1.40, 8.47)	0.007	2.99 (1.21, 7.39)	0.018
		Q3	3.29 (1.31, 8.24)	0.011	2.43 (0.96, 6.15)	0.061
		Q4	6.27 (2.60, 14.89)	<0.001	3.93 (1.61, 9.60)	0.003
		p for trend	<0.001		0.006	
	Body mass index	Q1	(reference)		(reference)	
		Q2	1.47 (0.66, 3.28)	0.344	1.39 (0.60, 3.00)	0.481
		Q3	2.75 (1.34, 5.63)	0.006	2.33 (1.12, 4.86)	0.023
		Q4	3.19 (1.56, 6.35)	0.001	2.32 (1.11, 4.87)	0.025
		p for trend		<0.001		0.009

a Adjusted for age, smoking status, alcohol drinking behavior, regular exercise, hypertension, increased low-density lipoprotein cholesterol, decreased high-density lipoprotein cholesterol, hypertriglyceridemia, hyperuricemia, diabetes mellitus, urine occult blood, hemoglobin and preserved eGFR at baseline. Cox proportional-hazards models were used to calculate hazard ratios and 95% confidence intervals. Definitions of these confounding factors are shown in Table 1.

CI, confidence interval; Q1, lowest quartiles; Q2, second quartiles; Q3, third quartiles; Q4, highest quartiles.

We also investigated the association between gender, WC, BMI and the incidence of CKD. In the unadjusted analyses, a graded relationship existed between each WC and BMI, and the risk of CKD in both gender. However, after adjusting for potential confounders, WC was not associated with the incidence of CKD in males.

### The Performance of the WheiR for Predicting CKD

**Figure 1:**
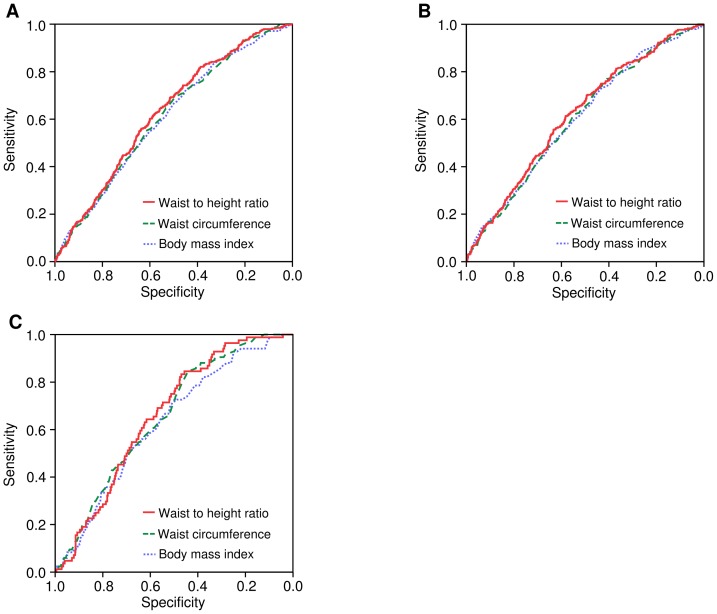
Receiver operating characteristic analyses describing the predictive value of each surrogate marker of obesity. (A) The area under the receiver operating characteristic curve (AUC) of waist to height ratio (WheiR), body mass index (BMI) and waist circumference (WC) were calculated as 0.628 (95% Confidence interval; 0.601–0.655), 0.611 (0.584, 0.638), and 0.607 (0.579, 0.635), respectively in all study participants. (B) and (C) Participants were stratified by gender. The AUCs of WheiR, BMI and WC were 0.619 (0.587, 0.650), 0.604 (0.572, 0.635), and 0.604 (0.572, 0.636), respectively, in male gender (B), and 0.660 (0.610, 0.710), 0.656 (0.604, 0.708), and 0.628 (0.572, 0.685), respectively, in female gender (C).

Finally, we performed the ROC analyses to examine which surrogate markers of body fat including WheiR, WC and BMI were superior predictors of the incidence of CKD. As shown in figure 1A, The AUC of WheiR was higher than those of both WC and BMI (WheiR, WC and BMI: 0.628 (95% Confidence interval; 0.601–0.655), 0.611 (0.584, 0.638), p = 0.015, and 0.607 (0.579, 0.635), p = 0.012, respectively). Figure 1B and 1C shows the gender difference of predicting values for the incidence of CKD. The AUC of WheiR was similar to those of both WC and BMI (WheiR, WC and BMI: 0.619 (0.587, 0.650), 0.604 (0.572, 0.635), p = 0.117, and 0.604 (0.572, 0.636), p = 0.052, respectively.) in male gender (Figure 1B), and the AUC of WheiR was also similar to those of both WC and BMI (WheiR, WC and BMI: 0.660 (0.610, 0.710), 0.656 (0.604, 0.708), p = 0.060, and 0.628 (0.572, 0.685), p = 0.604, respectively) in female gender (Figure 1C).

## Discussion

In this study, we found that the WheiR was an independent predictor for the incidence of CKD in the Japanese workers who participated. A graded relationship existed between quartiles of WheiR and the incidence of CKD. Compared with the lowest quartile, the incidence of CKD was 1.6 times higher in the highest quartile. WheiR had a strong predictive value for the incidence of both proteinuria and low eGFR. However, these relationships were abolished after adjustment BMI. There was a significant gender difference in the relationship between CKD and WheiR. After subdivision according to gender, the relationship between WheiR and the incidence of CKD was statistically significant in the unadjusted model in both genders. However, after adjusting for potential confounders including BMI, WheiR was significantly associated with the incidence of CKD in females, whereas it was not significant in males.

It is well-established that overall and central obesity are associated with cardiovascular risk factors such as hyperglycemia, diabetes, hypertension, dyslipidemia and metabolic syndrome [21], [22]. WheiR, WC and BMI could similarly predict cardiometabolic risk factors [22]. Most public health literature is focused on the use of BMI to identify obesity. However, BMI does not distinguish between muscle and fat, visceral and subcutaneous fat, or peripheral and central adiposity. A previous study indicated that WC strongly correlated with visceral fat as measured by computed tomography [23]. Furthermore, because height is also one of the factors that influences fat accumulation and distribution, WheiR might be a more accurate way to track fat distribution and accumulation than weight circumference [18]. Thus, we used WheiR as an index for central obesity.

Obesity and metabolic syndrome are known as independent risk factors for CKD [24], [25]. In previous studies, obesity has been implicated in the development focal segmental glomerulosclerosis and glomerulomegaly [26], [27]. Visceral adipose tissue secretes inflammatory cytokines such as interleukin-6 and tumor necrosis factor α, which are associated with both obesity-related glomerulopathy and metabolic syndrome [28]–[32]. It is not clear if it is central obesity or the related cardiometabolic risk factors that initiate renal injury. In the current study, the association between the WheiR and CKD remained robust even after adjusting for confounding factors, including cardiometabolic risk factors. This result suggested that the risk for CKD was not solely attributable to metabolic syndrome, which is consistent with a previous study that showed WheiR was significantly associated with CKD, independent of hypertension and diabetes [33].

In our results, WheiR was an independent predictor for the incidence of CKD in participants with eGFR < 80 mL/min/1.73 m^2^, however, this association was not significant in participants with eGFR ≥80 mL/min/1.73 m^2^. We speculated that this result was affected by inclusion of participants at different degree of proteinuria. Although several participants were diabetic, dyslipidemic and hypertensive in our study, we did not evaluate the microalbuminuria and had no information concerning the presence of proteinuria or kidney diseases without the results of self-administrated questionnaire before the study began. The lack of these data partially affected our results of the association between WheiR and the future appearance of a renal dysfunction.

Our results indicated the WheiR was an independent risk factor for the incidence of both low eGFR and proteinuria. However, the endpoint for the incident of proteinuria might lack the statistical and biological power, because p values were at the threshold of significance. Dipstick urinalysis might not be a reliable screening method for proteinuria. Previous study reported that the sensitivity, specificity and positive and negative predictive values of the dipstick test for detection of protein were 80.0%, 95.0%, 22.2% and 99.6% [34].

We found gender differences in the association between WheiR and the risk of CKD. Previous studies have reported that WheiR was more frequently associated with renal injury in females than males [33], [35]. Although it remains unclear how gender influences the correlation between WheiR and CKD, several hypotheses have proposed. First, the lack of an association between the WheiR and CKD in males suggested that the risk factors might be different between males and females and that factors other than central obesity might be more important in males. Second, anemia might contribute to the progression of CKD, especially in females. Because hypoxia can be exacerbated by lower hemoglobin levels, anemia might have a role in the progression of CKD. In our participants, the hemoglobin levels were lower values in females than those in males through the quartiles of WheiR (Table 2). However, in our study, after adjustment for potential confounders including hemoglobin, WheiR was still an independent predictor for CKD. Finally, another possibility is that sex hormones might have a role in the interaction with this association. Further studies are necessary to elucidate the mechanism by which gender differences have an influence on these relationships.

In this study, we also evaluated the predictive values of other markers of obesity. Our results indicated that BMI also had a strong predictive value as same as WheiR (Table 4, 7). Interestingly, as shown in Table 6, WheiR was still an independent risk factor for the incidence of CKD after adjustment for potential confounder including BMI in only females. This finding supported the gender differences in the association between WheiR and the risk of CKD.

The current study has several limitations. First, we did not evaluate the changes in WheiR in individual participants during the study period. Second, self-reported information regarding daily regular exercise, alcohol intake and smoking status is prone to underreporting, and misreporting could be a source of bias. Third, we did not investigate whether participants underwent kidney biopsy, thus, the etiology of the CKD associated with WheiR was not identified. However, the proportion of hypertensive and diabetes in participant with developing CKD were higher than those in participants without developing CKD (data not shown). These associations suggested that some of the etiology of CKD might be glomerular sclerosis and/or diabetic nephropathy. Fourth, we could not completely exclude the participants who had any infections, malignances or other peculiar disorders, because our self-administered standardized questionnaire did not require filling out about these information. Finally, there might be selection bias related to the healthy worker effect.

In conclusion, WheiR, which is one of the markers of central obesity, has the potential to be a useful surrogate marker for predicting the future development of CKD. There was a significant gender difference in the relationship between CKD and WheiR. Further study is needed to clarify the efficacy of interventions reducing the WheiR to decline the risk of CKD.
